# A phase 2, open-label study of ibrutinib plus rituximab in Japanese patients with Waldenstrom’s macroglobulinemia

**DOI:** 10.1007/s12185-024-03761-9

**Published:** 2024-04-10

**Authors:** Koji Izutsu, Hisashi Kato, Naohiro Sekiguchi, Tomoaki Fujisaki, Toshiro Kawakita, Naoshi Obara, Kosei Matsue, Mitsutaka Nishimoto, Tomoyoshi Hatayama, Mitsuo Inagaki, Ei Fujikawa

**Affiliations:** 1 Department of Hematology, National Cancer Center Hospital, 5-1-1 Tsukiji, Chuo-Ku, Tokyo, 104-0045 Japan; 2 Department of Hematology and Oncology, Osaka University Hospital, Osaka, Japan; 3 Division of Hematology, National Hospital Organization Disaster Medical Center, Tokyo, Japan; 4 Department of Internal Medicine, Matsuyama Red Cross Hospital, Matsuyama, Japan; 5 Department of Hematology, National Hospital Organization Kumamoto Medical Center, Kumamoto, Japan; 6 Department of Hematology, University of Tsukuba Hospital, Tsukuba, Japan; 7 Department of Hematology, Kameda General Hospital, Kamogawa, Japan; 8 Department of Hematology, Graduate School of Medicine, Osaka Metropolitan University, Osaka, Japan; 9 Research and Development Division, Janssen Pharmaceutical K.K, Tokyo, Japan

**Keywords:** Ibrutinib, Rituximab, Waldenstrom’s macroglobulinemia, Japanese patients

## Abstract

Ibrutinib is a first-in-class Bruton kinase inhibitor against B-cell neoplasms including Waldenström macroglobulinemia (WM). This study evaluated the efficacy and safety of ibrutinib-rituximab in Japanese patients with WM. Patients received ibrutinib 420 mg orally once daily plus weekly rituximab 375 mg/m^2^ IV (8 infusions total). The primary end point was major response rate (MRR; PR or better) by Independent Review Committee assessment. Secondary endpoints were progression-free survival (PFS), safety, pharmacokinetics, and biomarkers. Primary analysis was conducted in 16 patients [baseline, treatment naïve: 8 (50.0%); relapsed/refractory WM: 8 (50.0%)] who received ibrutinib-rituximab, after all patients completed Week 57 or end of treatment. At primary analysis, MRR was 87.5% [14/16 patients; 95% CI: 61.7, 98.4%; *p* < 0.0001 (null hypothesis: 32% response rate)]. At final analysis (median study intervention duration: 34.4 months, median follow-up: 35.0 months), MRR was unchanged at 87.5%, but VGPR [6/16 (37.5%)] and PR [8/16 (50.0%)] improved. Prior treatment status did not affect response. At final analysis, median PFS was not reached [36-month PFS rate: 86% (95% CI: 55, 96%)]. No critical safety signals were reported. This study demonstrated a positive benefit/risk profile of ibrutinib-rituximab in Japanese patients with WM, consistent with the iNNOVATE study.

**Supplementary Information:**

The online version contains supplementary material available at 10.1007/s12185-024-03761-9.

## Introduction

Waldenström macroglobulinemia (WM) is an indolent B-cell lymphoma characterized by the presence of lymphoplasmacytic lymphoma (LPL) in the bone marrow and accompanied by immunoglobulin type M (IgM) monoclonal gammopathy [1]. Clinical manifestations of WM are attributable to the IgM paraprotein, overall disease burden, and constitutional symptoms [2].

Prevalence of WM is higher in males, Caucasians, and in the elderly (≥65 years of age) [3]. In Japan, the proportion of patients with WM and LPL (229 and 125 cases, respectively) account for 1.97% of 17,957 mature lymphoid malignancies [4]. The annual incidence of WM/LPL in Japan was reported as 2.8 per million [4].

The currently available treatment options for treatment naïve or relapsed/refractory WM include alkylating agents (eg, cyclophosphamide or bendamustine), fludarabine, rituximab, bortezomib; and newer targeted therapies such as Bruton Tyrosine Kinase (BTK) inhibitors (e.g., tirabrutinib, ibrutinib), which is used as monotherapy or combination therapy [1, 5].

Ibrutinib, a potent and selective inhibitor of BTK, suppresses the downstream signaling pathway and induces the apoptosis of the cells [6]. The clinical benefit of ibrutinib has been demonstrated in global clinical trials. Activity of ibrutinib as monotherapy was demonstrated in the single-arm phase 2 study (PCYC-1118E) and its long term follow up [7, 8]. Moreover, the results from iNNOVATE phase 3 trial demonstrated that ibrutinib-rituximab combination resulted in longer progression-free survival (PFS) than placebo-rituximab in patients with treatment-naïve and relapsed WM [9]. Ibrutinib is approved for the treatment of WM in many countries including United States (US) and European Union (EU) either as a single agent or combination therapy with rituximab, based on the results of these trials [5], and is listed as an option for symptomatic WM in National Comprehensive Cancer Network [NCCN] and European Society for Medical Oncology [ESMO] guidelines [5, 10].

As the previous global trials did not include Japanese patients with WM, the present study intended to evaluate the efficacy, safety, pharmacokinetics (PK) and biomarker analysis of ibrutinib in combination with rituximab in Japanese patients with treatment naïve or relapsed/refractory WM.

## Materials and methods

### Study design and treatment

This was an open-label, single arm, multicenter, phase 2 study conducted at 9 sites in Japan that evaluated the efficacy and safety of ibrutinib plus rituximab in patients with treatment naïve or relapsed/refractory WM (study period: 25 September 2019 to 2 March 2023). Patients received oral ibrutinib 420 mg daily until confirmed disease progression or unacceptable toxicity or any other criteria for permanent discontinuation were met, along with IV rituximab 375 mg/m^2^ weekly for 4 consecutive weeks, followed by a second course of IV rituximab administered weekly for 4 consecutive weeks after a 12-week interval with a total of 8 infusions. Patients who completed EOT visit due to discontinuation of the study for reasons other than progressive disease (PD) were followed every 12 weeks for the assessment of overall disease, survival status and new anticancer therapy use until progressive disease or use of alternative anticancer therapy. Patients who discontinued study intervention due to disease progression were followed for approximately every 12 weeks to assess survival status, the use of alternative antineoplastic therapy, and occurrence of any other malignancy until death, withdrawal of consent by patients, lost to follow-up, or study terminated by sponsor, whichever occurred first.

The study protocol was approved by the local Institutional Review Board and the study was conducted in accordance with the ethical principles originating in the Declaration of Helsinki, the International Conference on Harmonization Good Clinical Practice guidelines, applicable regulatory requirements, and in compliance with the protocol. All participating patients provided written informed consent to participate in the study.

### Patients

Eligible patients were Japanese ≥20 years of age with clinicopathological diagnosis of WM in accordance with the consensus panel of the second IWWM, central pathologic review was performed to confirm clinicopathological diagnosis; patients with treatment naïve or relapsed/refractory WM having a measurable disease (defined as serum monoclonal IgM  >0.5 g/dL); having a symptomatic disease meeting at least 1 of the recommendations from the second IWWM [11] for requiring treatment; and with an ECOG performance status of ≤2. The patients with following clinical criteria were excluded from the study: with known histologic involvement of the central nervous system by WM, received rituximab treatment within the last 12 months before the first dose of study intervention and plasmapheresis  <35 days prior to the initiation of study intervention; who had prior exposure to ibrutinib or other BTK inhibitors; received any WM-related therapy ≤30 days prior to first administration of study intervention; history of stroke or intracranial hemorrhage within 12 months prior to enrollment, or with clinically significant cardiovascular disease, or known bleeding disorder.

### Endpoints and assessments

The primary endpoint was major response rate (MRR) evaluated by IRC assessment (PR or better is defined as overall response in the study protocol originally). The secondary endpoints were progression-free survival (PFS) assessed by IRC assessment, safety, pharmacokinetics, and biomarker analysis. Time to response (TTR), duration of response (DOR), sustained Hgb improvement, overall survival (OS) and time to next treatment (TTNT) were also evaluated as exploratory endpoints. The primary analysis for all efficacy and safety endpoints was performed when all patients completed the assessment of Week 57 or EOT visit, whichever occurred earlier. After the primary analysis, this study was closed by sponsor following approval for the WM indication in Japan. Final analysis was conducted at the end of study, when all the patients have completed all planned assessments or discontinued the study. Here we present efficacy results for primary analysis and final analysis. Safety results are presented for final analysis follow up. The MRR was defined as the proportion of patients who achieved a best response of confirmed CR, VGPR, or PR (ie, ≥PR). Response was defined by the modified sixth IWWM [12]. PFS was the duration from treatment initiation to disease progression or death, whichever occurred first. TTR was duration from treatment initiation to initial documentation of an IRC assessed response (≥PR) in responders. DOR was duration from initial documentation of an IRC assessed response (≥PR), to first documented evidence of PD, or death, whichever occurred first. Sustained Hgb improvement was defined as the hemoglobin improvement (increase to  >11 g/dL with an increase of ≥0.5 g/dL over baseline for patients with baseline is ≤11 g/dL or increase of  ≥2 g/dL over baseline) continuously for ≥56 days (8 weeks) without blood transfusion. OS was defined as duration of treatment initiation to death due to any cause. TTNT was duration from treatment initiation to the start of any subsequent systematic therapy for WM. Safety assessment included TEAEs defined as any AE occurring at or after the initial administration of ibrutinib plus rituximab through the day of last dose plus 30 days was treatment emergent. All reported TEAEs were included in the analysis summarized by system organ class and preferred term. Death, serious TEAEs, and TEAEs leading to discontinuation were also summarized. PK parameters of ibrutinib and its metabolite evaluated were C_max_, AUC_0–24_, AUC_last_, and T_max_, assessed at Week 4/day 1. For biomarker analysis the prognostic biomarkers relative to disease and/or treatment outcomes evaluated were MYD88, and CXCR-4 mutational status. DNA was extracted unselected bone marrow samples, and gene mutational analysis was performed by next generation sequencing (NGS method) using an Ion AmpliSeq HD Library Kit.

### Statistical analyses

A target sample size of 14 patients were planned to be analyzed in efficacy analysis to demonstrate that the lower limit of exact 2-sided 95% CI of estimated MRR exceeded 32% with 80% power, assuming an expected MRR of 72%. The assumptions for sample size calculation were based on the previous study (iNNOVATE) results, where the response rate was 72% for the ibrutinib plus rituximab arm, and 32% for the placebo plus rituximab arm.

## Results

### Patients

A total of 21 patients were screened, out of which 16 were enrolled and included in the analysis. All enrolled patients were treated with at least 1 dose of study intervention. At the time of primary analysis data cut-off (24 August 2021), all the treated patients completed the treatment with rituximab and 12 (75.0%) were still on treatment with ibrutinib. At final analysis, 5 (31.3%) discontinued the treatment and 11 (68.8%) patients were on-treatment with ibrutinib and regarded as completed. The reasons for discontinuation of ibrutinib were refusal of patients [3 (18.8%)], progressive disease and other (due to moving) [1 (6.3%)]. Patient disposition is presented in **Supplementary Figure 1**.

The median (range) age of patients was 68.0 (ranged from 39 to 84) years, and 12 (75.0%) were male. In total, 8 patients (50.0%) were treatment naïve, and 8 (50.0%) were with relapsed/refractory WM (7 had received 1 to 2 prior WM-related therapies). The median (range) time from initial diagnosis to first dose of ibrutinib for treatment naïve patients and those with relapsed/refractory WM was 2.4 (0.5–41.4) months and 95.0 (61.9–221.1) months, respectively. In relapsed/refractory patients, the median (range) time from the last prior treatment to first ibrutinib treatment was 52.4 (15.7–112.7) months. The most common criteria for initiating treatment of WM at screening were symptomatic anemia (62.5%), clinically relevant fatigue (50.0%), and symptomatic hyper-viscosity (18.8%). Majority of patients had median IgM, 36.6 g/L, β2-microglobulin level >3 mg/L (68.8%), hemoglobin ≤110 g/L (75.0%). Median monoclonal protein spike was 22.0 g/L. The baseline demographics, clinical characteristics in Table 1 and symptoms for WM are presented in **Supplementary Table 1**. Among the patients who received prior WM-related systemic therapies, majority received rituximab [7/8 (87.5%) patients]. Among these, 62.5% used rituximab in combination with other antineoplastic agents (doxorubicin hydrochloride, melphalan hydrochloride, bendamustine hydrochloride, cyclophosphamide hydrate).

**Table 1:** Summary of demographic and baseline characteristics

Characteristic	Ibrutinib plus rituximab
N	16
Age, mean (SD)	67.5 (11.2)
Range	39; 84
>65 years	11 (68.8%)
>75 years	3 (18.8%)
Sex	
Female	4 (25.0%)
Male	12 (75.0%)
Serum IgM (g/L), mean (SD)	36.9 (22.3)
Range	6.0; 77.1
>70	1 (6.3%)
Beta-2 microglobulin (mg/L), mean (SD)	4.2 (2.0)
Range	1.7; 9.7
>3	11 (68.8%)
Months from initial diagnosis to first dose of ibrutinib, mean (SD)	60.2 (62.1)
Treatment naïve, mean (SD)	12.2 (16.1)
Relapsed/refractory, mean (SD)	108.2 (52.3)
ECOG performance status, n (%)	
0	11 (68.8%)
1	5 (31.3%)
2	0
Number of prior therapies, n (%)	
0	8 (50.0%)
1	5 (31.3%)
2	2 (12.5%)
≥3	1 (6.3%)
IPSS-WM risk category, n (%)	
Low	4 (25.0%)
Intermediate	4 (25.0%)
High	8 (50.0%)
MYD88L265P/CXCR4WHIM mutation status (Bone marrow)^a^	
N	7
MYD88^L265P^/CXCR4^WT^	4 (57.1%)
MYD88^L265P^/CXCR4^WHIM^	2 (28.6%)
MYD88^WT^/CXCR4^WT^	1 (14.3%)

Percent is calculated using “N” of each item

*IGM* Immunoglobulin M, *SD* standard deviation, *WM* Waldenstrom’s macroglobulinemia

^a^7 samples (patients) were evaluable for biomarker analysis for MYD88^L265P^ and CXCR4^WHIM^

### Efficacy

#### Response

**Fig. 1:**
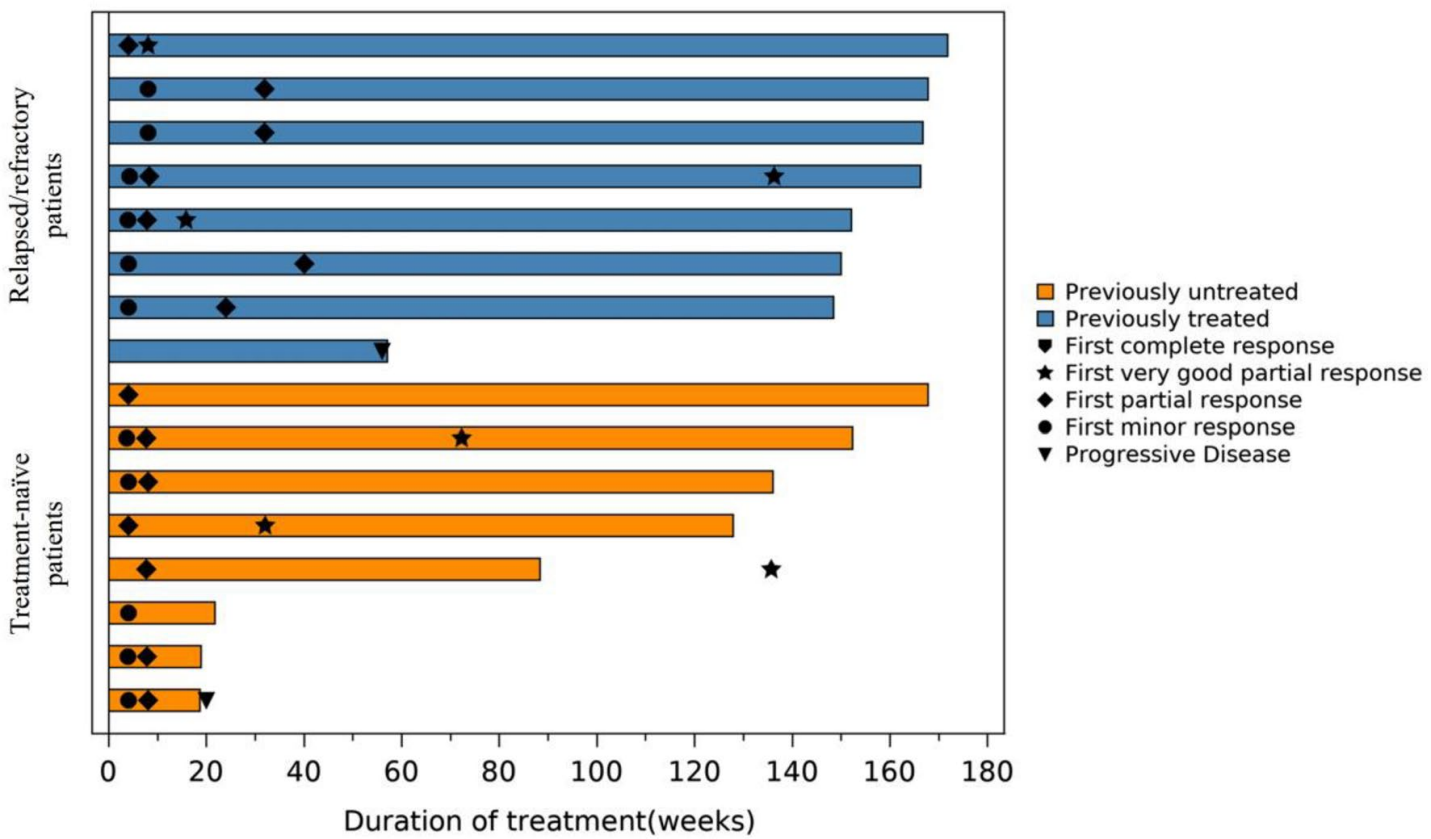
Duration of treatment and responses. A swimmer plot that presents the duration of treatment, the first timings of responses, and the timing of progressive disease for each patient

At primary analysis, the median duration of study intervention was 16.6 months (range: 4.3; 22.0) and it was 34.3 months (range: 4.3; 39.6) at final analysis. The duration of treatment and responses are presented in Fig. 1. At primary analysis, the MRR per IRC for ibrutinib plus rituximab was 87.5% (14/16 patients; 95% CI: 61.7, 98.4%; *p* < 0.0001) (Table 2 represents data from final analysis) which was unchanged at 87.5% until final analysis. At final analysis, among the patients who achieved the response, confirmed VGPR was reported in 6/16 (37.5%) and PR in 8/16 (50.0%) patients. Minor response was reported by 1 (6.3%) patient and there was one patient who was not evaluable for the best response per the IRC charter, as evaluable post-treatment images were not available. The MRR reported in previously treated patients with relapsed/refractory WM was the same as that reported in patients who were treatment naïve (87.5% in each) (Table 2). Although it was difficult to evaluate the relationship between IPSS-WM and efficacy because of the small sample size, IPSS-WM in the 2 patients who did not achieve a major response was rated as intermediate (not evaluable patient) and high (minor response patient). At final analysis, in patients who achieved PR or better, the median TTR was 1.87 months (range: 1.0–14.7 months), and the median DOR was not reached.

**Table 2:** Summary of primary efficacy, final analysis (response evaluable analysis set)

Parameters	Ibrutinib plus rituximab N = 16
Best response	
Complete response (CR)	0
Very good partial response (VGPR)	6 (37.5%)
Partial response (PR)	8 (50.0%)
Minor response (MR)	1 (6.3%)
Stable disease (SD)	0
Progressive disease (PD)	0
Not evaluable/not applicable	1 (6.3%)
Major response rate (CR, VGPR, PR)*	
N (%) [95% CI^a^]; *P* value^b,c^	14 (87.5%) [61.7%, 98.4%]; <0.0001
	Responder/N of Subgroup (MRR)^d^
MRR by prior WM therapy, n/N (%)	
Previously Untreated	7/8 (87.5%)
Previously Treated	7/8 (87.5%)

*CI* confidence interval; *WM* waldenstrom’s macroglobulinemia

^*^Originally the response of PR or better is defined as overall response in the study protocol

^a^95% CI is calculated using Clopper-Pearson’s exact method

^b^One-sided *p*-value calculated based on the null hypothesis of 32% response rate and exact binomial distribution

^c^At final analysis the *P* value is nominal

^d^Denominator is the number of subjects in the subgroup

#### Progression-free survival

**Fig. 2:**
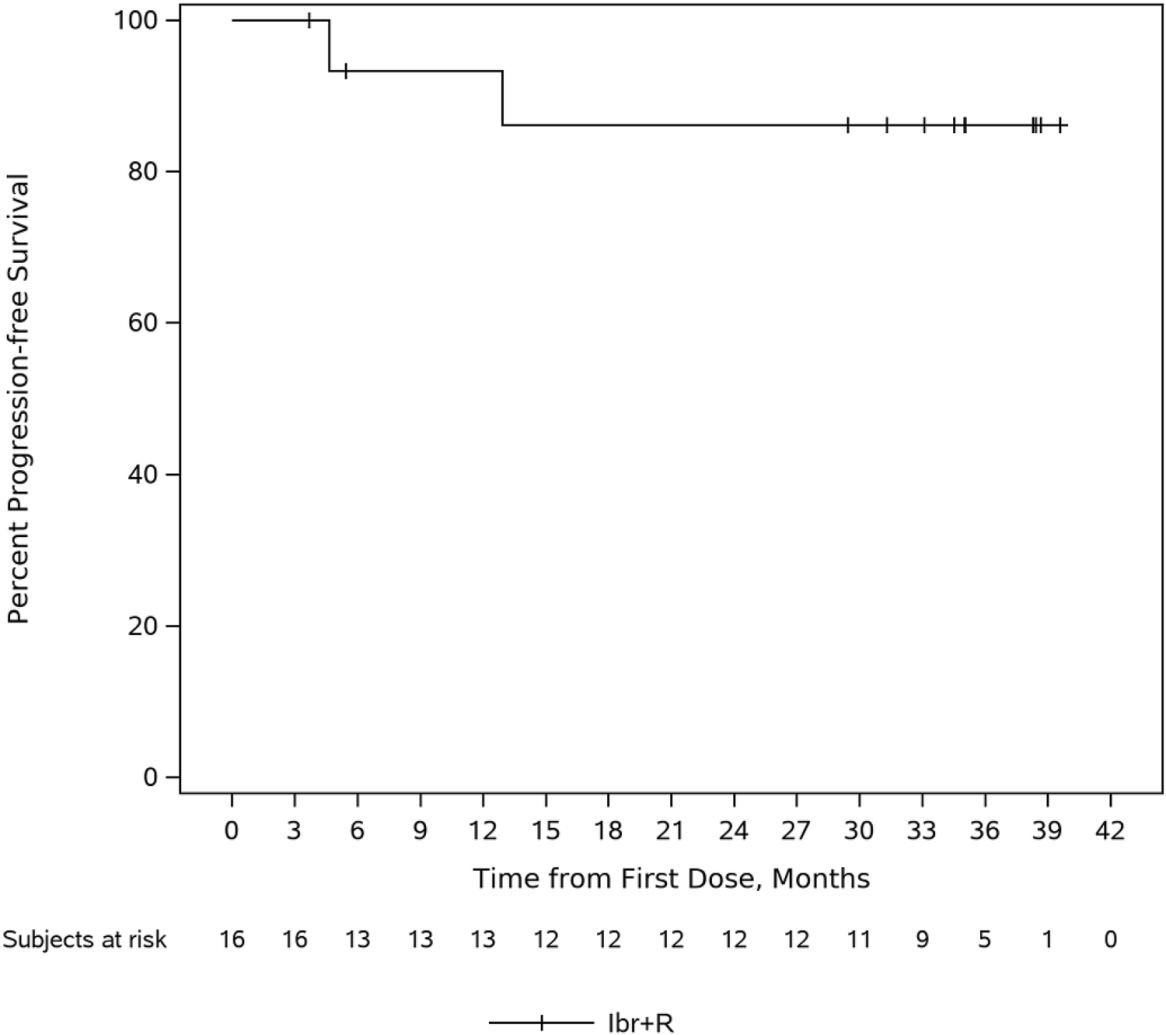
Progression-free survival. *Ibr + R* ibrutinib + rituximab

At final analysis, with median follow up of 35.0 months, median PFS based on the Kaplan–Meier estimate was not reached. The PFS rate was 86% (95% CI: 55, 96%) at 36-months (Fig. 2). At primary analysis, among 16 patients, 2 (12.5%) had progressive disease. One patient had progressive disease during the treatment period which led to discontinuation, and the other patient had progressive disease during follow-up period after refused further treatment. At final analysis, there were no additional patients with progressive disease.

#### Sustained hemoglobin improvement and change of serum immunoglobulin M from baseline

The proportion of patients with sustained hemoglobin improvement was 68.8% at primary analysis which was increased to 75.0% at final analysis. Among the 16 patients, 12 had baseline hemoglobin ≤11 g/dL and out of these 11 (91.7%) achieved sustained hemoglobin improvement, at final analysis. At baseline, the mean (SD) IgM level was noted to be high at 36.9 (±22.3) g/L which reduced to 13.1 (±16.3) g/L at primary analysis and 8.0 (±7.6) g/L at final analysis. At final analysis, for 13 evaluable patients, the mean (±SD) change from baseline over time in IgM level was  −28.2 (±19.2) g/L.

#### Overall survival and time to next treatment

**Fig. 3:**
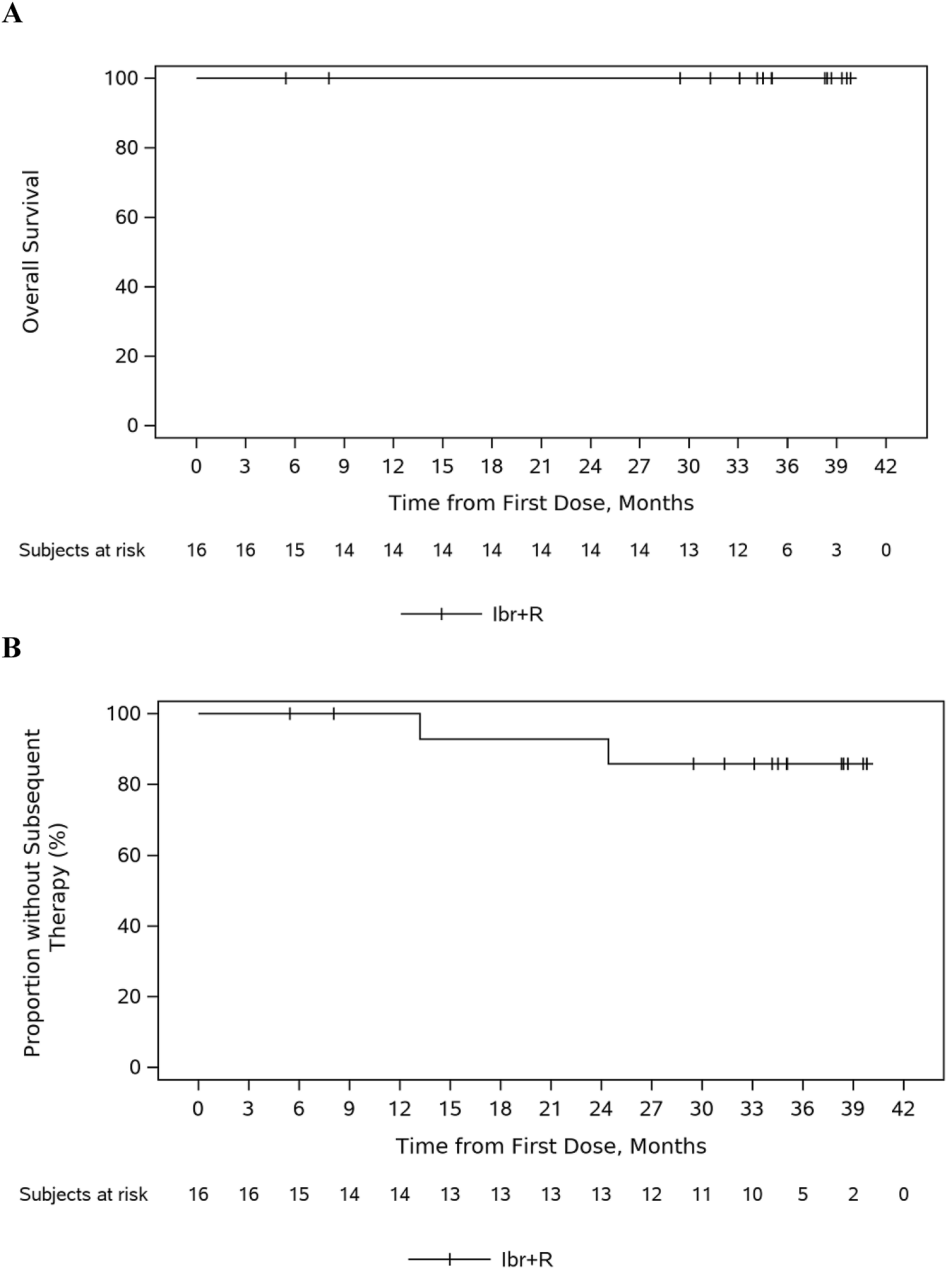
**A** Overall survival and **B** Time to next treatment. *Ibr + R* ibrutinib + rituximab

At final analysis, with median follow-up of 35.0 months, the median OS based on the Kaplan–Meier estimate was not reached; all 16 (100%) patients remained alive at final analysis (Fig. 3A). The median TTNT based on the Kaplan–Meier estimate for all the patients (N = 16) was not reached. At final analysis, 2 (12.5%) patients had received subsequent systematic therapies for WM (1 patient had received rituximab, bendamustine and dexamethasone followed by tirabrutinib hydrochloride, and another 1 had received rituximab, study drug) (Fig. 3B).

#### Safety

At final analysis, all 16 patients experienced at least 1 TEAE. TEAEs considered to be related to study intervention were reported in 13 (81.3%) patients. In total, 12 (75.0%) patients reported ibrutinib-related TEAEs and 9 (56.3%) reported rituximab-related TEAEs. There were no TEAEs leading to discontinuation or death. Safety summary is presented in Table 3.

**Table 3:** Summary of safety, final analysis (safety analysis set)

	Ibrutinib plus rituximab N = 16
TEAEs	16 (100.0%)
Grade ≥3	12 (75.0%)
TEAEs related to drug^a^	13 (81.3%)
Grade ≥3	9 (56.3%)
TEAEs related to ibrutinib^a^	12 (75.0%)
Grade ≥3	7 (43.8%)
TEAEs related to rituximab^a^	9 (56.3%)
Grade ≥3	3 (18.8%)
Serious TEAEs	4 (25.0%)
Grade ≥3	4 (25.0%)
Serious TEAEs related to drug^a^	1 (6.3%)
Grade ≥3	1 (6.3%)
Serious TEAEs related to ibrutinib^a^	1 (6.3%)
Grade ≥3	1 (6.3%)
Serious TEAEs related to rituximab^a^	0
Grade ≥3	0
TEAEs leading to death^b^	0
TEAEs leading to discontinuation of ibrutinib	0
TEAEs leading to discontinuation of rituximab	0
TEAEs leading to dose reduction of ibrutinib	2 (12.5%)
Grade ≥3	2 (12.5%)
TEAEs leading to interruption of ibrutinib	11 (68.8%)
Grade ≥3	7 (43.8%)
TEAEs leading to discontinuation of rituximab	6 (37.5%)
Grade ≥3	2 (12.5%)

*TEAE* treatment emergent adverse event

^a^An TEAE is categorized as related if assessed by the investigator as possibly, probably, or very likely related to study drug

^b^TEAEs leading to death are based on AE outcome of Fatal

The most frequently (>15%) reported TEAEs were rash reported in 6 (37.5%) patients; neutrophil count decreased, pyrexia and hypertension in 5 (31.3%) patients each; contusion in 4 (25.0%) patients; and platelet count decreased, white blood cell count decreased, nasopharyngitis, erythema, pruritus, abdominal pain upper, and diarrhea in 3 (18.8%) patients each (Table 4). The most frequently reported (>15%) TEAEs related to ibrutinib were atrial fibrillation and hypertension reported in 4 (25.0%) each; neutrophil count decreased reported in 5 (31.3%), and platelet count decreased reported in 3 (18.8%) patients each.

**Table 4:** TEAEs occurring with ≥10% frequency, final analysis (safety analysis set)

	Ibrutinib plus rituximab	
	All patients (N = 16)	Grade ≥3 (N = 16)
Subjects with ≥1 TEAE	16 (100.0%)	12 (75.0%)
Rash	6 (37.5%)	0
Neutrophil count decreased	5 (31.3%)	2 (12.5%)
Hypertension	5 (31.3%)	4 (25.0%)
Pyrexia	5 (31.3%)	1 (6.3%)
Atrial fibrillation	4 (25.0%)	0
Contusion	4 (25.0%)	0
COVID-19	3 (18.8%)	1 (6.3%)
Nasopharyngitis	3 (18.8%)	0
Erythema	3 (18.8%)	0
Pruritus	3 (18.8%)	0
Abdominal pain upper	3 (18.8%)	0
Diarrhea	3 (18.8%)	0
Platelet count decreased	3 (18.8%)	0
White blood cell count decreased	3 (18.8%)	0
Cystitis	2 (12.5%)	0
Paronychia	2 (12.5%)	0
Viral infection	2 (12.5%)	0
Abdominal discomfort	2 (12.5%)	0
Constipation	2 (12.5%)	0
Mouth hemorrhage	2 (12.5%)	0
Aspartate aminotransferase increased	2 (12.5%)	0
Lymphocyte count decreased	2 (12.5%)	1 (6.3%)
Arthralgia	2 (12.5%)	0
Back pain	2 (12.5%)	0
Oedema peripheral	2 (12.5%)	0
Palpitations	2 (12.5%)	0
Oropharyngeal pain	2 (12.5%)	0
Headache	2 (12.5%)	0
Dry eye	2 (12.5%)	0
Dehydration	2 (12.5%)	2 (12.5%)
Prostatitis	2 (12.5%)	1 (6.3%)
Anaemia	2 (12.5%)	1 (6.3%)

*TEAE* treatment emergent adverse event

Subjects are counted only once for any given event, regardless of the number of times they actually experienced the event. Subjects with multiple events with grade ≥3 for a given preferred term, system organ class are counted once only with maximum severity for each category

The incidence of TEAEs from day 1 to Week 16 were reported in 15 (93.8%) patients, from Week 16 to Week 32 in 12 (75.0%) patients and after Week 32 in 12 (80.0%) patients. TEAEs reported by onset of timing (occurrence period) are presented in Table 5.

**Table 5:** TEAEs by occurrence period (in ≥10% patients), final analysis (safety analysis set)

	Ibrutinib plus rituximab			
	All patients (N = 16)	<16 Weeks (N = 16)	≥16 Weeks, <32 Weeks (N = 16)	≥32 Weeks (N = 15)
Subjects with ≥1 TEAE	16 (100.0%)	15 (93.8%)	12 (75.0%)	12 (80.0%)
Rash	6 (37.5%)	2 (12.5%)	2 (12.5%)	3 (20.0%)
Neutrophil count decreased	5 (31.3%)	3 (18.8%)	3 (18.8%)	3 (20.0%)
Hypertension	5 (31.3%)	0	2 (12.5%)	4 (26.7%)
Pyrexia	5 (31.3%)	2 (12.5%)	2 (12.5%)	2 (13.3%)
Atrial fibrillation	4 (25.0%)	1 (6.3%)	0	3 (20.0%)
Contusion	4 (25.0%)	0	2 (12.5%)	2 (13.3%)
COVID-19	3 (18.8%)	0	0	3 (20.0%)
Nasopharyngitis	3 (18.8%)	3 (18.8%)	1 (6.3%)	1 (6.7%)
Erythema	3 (18.8%)	3 (18.8%)	2 (12.5%)	0
Pruritus	3 (18.8%)	2 (12.5%)	1 (6.3%)	1 (6.7%)
Abdominal pain upper	3 (18.8%)	1 (6.3%)	1 (6.3%)	1 (6.7%)
Diarrhea	3 (18.8%)	0	2 (12.5%)	1 (6.7%)
Platelet count decreased	3 (18.8%)	3 (18.8%)	0	0
White blood cell count decreased	3 (18.8%)	1 (6.3%)	2 (12.5%)	2 (13.3%)
Cystitis	2 (12.5%)	1 (6.3%)	0	1 (6.7%)
Paronychia	2 (12.5%)	0	2 (12.5%)	1 (6.7%)
Viral infection	2 (12.5%)	2 (12.5%)	0	0
Abdominal discomfort	2 (12.5%)	1 (6.3%)	0	1 (6.7%)
Constipation	2 (12.5%)	2 (12.5%)	0	0
Mouth hemorrhage	2 (12.5%)	1 (6.3%)	0	1 (6.7%)
Aspartate aminotransferase increased	2 (12.5%)	1 (6.3%)	1 (6.3%)	1 (6.7%)
Lymphocyte count decreased	2 (12.5%)	2 (12.5%)	1 (6.3%)	1 (6.7%)
Arthralgia	2 (12.5%)	0	0	2 (13.3%)
Back pain	2 (12.5%)	0	0	2 (13.3%)
Oedema peripheral	2 (12.5%)	1 (6.3%)	1 (6.3%)	1 (6.7%)
Palpitations	2 (12.5%)	1 (6.3%)	0	1 (6.7%)
Oropharyngeal pain	2 (12.5%)	0	0	2 (13.3%)
Headache	2 (12.5%)	2 (12.5%)	1 (6.3%)	0
Dry eye	2 (12.5%)	1 (6.3%)	0	1 (6.7%)
Dehydration	2 (12.5%)	1 (6.3%)	1 (6.3%)	1 (6.7%)
Prostatitis	2 (12.5%)	0	0	2 (13.3%)
Anaemia	2 (12.5%)	1 (6.3%)	0	1 (6.7%)

*TEAE* treatment emergent adverse event

Subjects are counted only once for any given event, regardless of the number of times they actually experienced the event

The denominator is the number of subjects who are participating in the study during each time period

Grade ≥3 TEAEs were reported in 12 (75.0%) patients and the most frequently reported Grade ≥3 TEAEs were hypertension [4 (25.0%)], neutrophil count decreased and dehydration [2 (12.5%) in each]. Serious TEAEs were reported in 4 (25.0%) patients; serious TEAEs reported after primary analysis were arthritis bacterial, COVID-19, Escherichia bacteremia, pneumonia bacterial, hypophagia, and cystitis hemorrhagic [1 (6.3%) patient each]. All serious TEAEs were Grade ≥3 in severity except arthritis bacterial. For majority of serious TEAEs, the relationship with study intervention was considered as doubtful by the investigator except for pneumonia bacterial, COVID-19, dehydration and hypophagia which were considered not related with ibrutinib. The serious TEAE of cellulitis was considered as probably related to ibrutinib.

TEAEs leading to dose reduction of ibrutinib, and interruption of ibrutinib were reported in 2 (12.5%), and 11 (68.8%) patients, respectively (Table 3). IgM rebound due to interruption was observed in 5 patients. All these patients did not have withdrawal symptom, and serum IgM level of all patients were reduced after restart of the ibrutinib administration. A total of 6 (37.5%) patients had 1 or more TEAEs leading to dose interruption of rituximab during the study. Treatment with ibrutinib was interrupted due to all serious TEAEs except Escherichia bacteremia, arthritis bacterial and hypophagia. All the serious TEAEs were reported as resolved at the time of final analysis except cellulitis which was reported as recovering/resolving.

Among the TEAEs, rash was reported as related to ibrutinib and rituximab in 2 (12.5%) patients each. In total, 7 (43.8%) patients had  ≥1 rash events. The rash events reported were rash [6 (37.5%)], rash maculo-papular and genital rash [1 (6.3%) patients each]. There were no patients who reported Grade ≥3 TEAEs of rash.

Infusion related reactions were reported in 7 (43.8%) patients. The infusion related reaction reported were erythema [3 (18.8%) patients]; pruritus, rash, and pyrexia [2 (12.5%) patients]; headache, neuropathy peripheral, presyncope, supraventricular extrasystoles and eyelid oedema [1 (6.3%) patients]. Infusion-related reactions TEAEs of Grade  ≥3 were reported in 2 (12.5%) patients which were presyncope and pyrexia [1 (6.3%) patients each].

There were no patients with liver function abnormalities as per the Hy’s Law laboratory criteria, and IgM flare.

#### Pharmacokinetics

For ibrutinib (PK evaluable set, n = 15); at Week 4/Day 1; the mean (SD) C_max_ (n = 15) was 122 (149) ng/mL, AUC_24h_ (n = 12) was 763 (834), AUC_last_ (n = 15) was 836 (1115) ng*h/mL, and median T_max_ (n = 15) after first dose was 3.6 h (range: 0.8–4.2 h) (**Supplementary Table 2**). For PCI-45227 (PK evaluable set, n = 15); at Week 4/Day 1; the mean (SD) C_max_ (n = 15) was 87.2 (57.3) ng/mL, AUC_24h_ (n = 11) was 1152 (808) ng*h/mL, AUC_last_ (n = 15) was 1075 (768) ng*h/mL, and median T_max_ (n = 15) after first dose was 3.75 h (range: 1.75–5.03 h) (**Supplementary Table 2**).

The metabolite/parent ratio [mean (SD)] based on the C_max_ (n = 15), AUC_24h_ (n = 11), AUC_last_ (N = 15), were 1.22 (0.946), 1.78 (0.862), 3.01 (3.07), respectively.

### Biomarker analysis

Among the bone marrow samples from 10 of the 16 participants, 7 samples provided assay results, and 3 samples were not evaluable. MYD88^L265P^ mutations were present in 6/7 (85.7%). CXCR4^WHIM^ mutations were present in 2/7 (28.6%). The major response rates for each of the subgroups MYD88^L265P^/CXCR4^WT^, MYD88^L265P^/CXCR4^WHIM^, and MYD88^WT^/CXCR4^WT^ using the bone marrow samples were 4/4 (100.0%) 2/2 (100.0%) and 0/1 (0%), respectively.

## Discussion

This Phase 2, open-label study presents the efficacy, safety, and pharmacokinetics of ibrutinib plus rituximab in Japanese patients with WM. The outcome of the study demonstrates that among Japanese patients with treatment naïve or relapsed/refractory WM, ibrutinib in combination with rituximab shows rapid and high degree of clinical activity across all efficacy endpoints, along with an acceptable safety profile.

A high MRR of 87.5% was observed in Japanese patients regardless of prior treatment status which is comparable with the phase 3 iNNOVATE study where 76% MRR was reported in non-Japanese patient population treated with ibrutinib plus rituximab [9]. The findings with ibrutinib are consistent with the findings from studies with other BTK inhibitors in Japanese patient population [12, 13]. A phase 2 study of tirabrutinib monotherapy in Japanese patients reported that the MRR in relapsed/refractory and treatment naïve WM demonstrated similar response rates (CR + VGPR + PR) of 88.9 and 88.9%, respectively [14]. Another study with zanubrutinib monotherapy in Japanese patients with WM reported an MRR (CR + VGPR + PR) of 61.6 and 83.4% in treatment-naïve and relapsed/refractory WM, respectively [13].

In this study, with a median follow up of 35.0 months at final analysis, the median PFS was not reached which indicates longer PFS with ibrutinib plus rituximab. The PFS rate observed at 36-months was 86% which is comparable to the iNNOVATE study where longer PFS was reported with ibrutinib-rituximab than placebo-rituximab in patients with treatment-naïve and relapsed/refractory WM [9]. Also, similar to the iNNOVATE study, substantially more patients (~ 88%) did not receive any subsequent systematic therapy for WM. Most of the patients in the current study achieved sustained hemoglobin improvement which was similar to that reported in the iNNOVATE study [9].

There were no new safety signals observed in Japanese patients with WM treated with ibrutinib plus rituximab. At final analysis, all patients experienced at least 1 TEAE. Consistent with the previous studies [15–17] and the iNNOVATE study [9], the most common TEAEs of any grade and grade  ≥3 were similar in type and prevalence. The prevalence of TEAEs decreased overtime. The proportion of patients with grade  ≥3 and drug related TEAEs were 62.5% and about 50%, respectively. However, there were no TEAEs (any/grade ≥3) leading to death or discontinuation of either ibrutinib or rituximab. Also, fewer patients reported TEAEs leading to dose reduction of ibrutinib and dose interruption of ibrutinib and rituximab. Overall, these safety results are consistent with the iNNOVATE study [9] and indicate that ibrutinib plus rituximab was well tolerated in Japanese patients with WM. The sample size of the present study is small, and it is necessary to continue to investigate the efficacy and safety of ibrutinib in clinical practice.

In this study, the mean maximum plasma concentration (C_max_) of ibrutinib was 122 ng/mL, with a rapid absorption of about 3 h (median T_max_) and mean elimination half-life (t_1/2_) of approximately 4–5 h. The mean steady state AUC_24_ and AUC_last_ observed in patients on ibrutinib 420 mg were 763 and 836 ng*h/mL, respectively. When compared to the PK parameters observed in non-Japanese patients with WM there was no difference observed [9]. Also, the PK results were similar between the combination therapy of ibrutinib and rituximab and single agent ibrutinib dose of 420 mg in Japanese patients regardless of the treated disease [18].

## Conclusions

In summary, this study demonstrated a positive benefit/risk profile of ibrutinib plus rituximab in treatment of Japanese patients with treatment-naïve or relapsed/refractory WM.

### Supplementary Information

Below is the link to the electronic supplementary material.

Supplementary file1 (DOCX 74 KB)

## Data Availability

The data sharing policy of Janssen Pharmaceutical Companies of Johnson & Johnson is available at https://www.janssen.com/clinical-trials/transparency. As noted on this site, requests for access to the study data can be submitted through Yale Open Data Access (YODA) Project site at http://yoda.yale.edu.

## References

[1] McMaster ML (2023). The epidemiology of Waldenström macroglobulinemia. Semin Hematol.

[2] Pratt G, El-Sharkawi D, Kothari J, D’Sa S, Auer R, McCarthy H (2022). Diagnosis and management of Waldenström macroglobulinaemia-A British Society for Haematology guideline. Br J Haematol.

[3] Buske C, Jurczak W, Salem JE, Dimopoulos MA (2023). Managing Waldenström’s macroglobulinemia with BTK inhibitors. Leukemia.

[4] Sekiguchi N. [Waldenström macroglobulinemia: Japanese perception]. Rinsho Ketsueki. 2019;60(8):988–97. Japanese. 10.11406/rinketsu.60.988. PMID: 31484900. 10.11406/rinketsu.60.988 31484900

[5] Kastritis E, Leblond V, Dimopoulos MA, Kimby E, Staber P, Kersten MJ, et al. Waldenstrom’s macroglobulinaemia: ESMO Clinical Practice Guidelines for diagnosis, treatment and follow-up. Ann Oncol. 2018;29(Suppl 4):iv41–iv50. 10.1093/annonc/mdy146 29982402

[6] Yang G, Zhou Y, Liu X, Xu L, Cao Y, Manning RJ (2013). A mutation in MYD88 (L265P) supports the survival of lymphoplasmacytic cells by activation of Bruton tyrosine kinase in Waldenström macroglobulinemia. Blood.

[7] Treon SP, Tripsas CK, Meid K, Warren D, Varma G, Green R (2015). Ibrutinib in previously treated Waldenström’s macroglobulinemia. N Engl J Med.

[8] Treon SP, Meid K, Gustine J, Yang G, Xu L, Liu X (2021). Long-Term follow-up of ibrutinib monotherapy in symptomatic, previously treated patients with Waldenström macroglobulinemia. J Clin Oncol.

[9] Buske C, Tedeschi A, Trotman J, García-Sanz R, Macdonald D, Leblond V (2022). Ibrutinib plus rituximab versus placebo plus rituximab for Waldenström’s macroglobulinemia: final analysis from the randomized phase III iNNOVATE study. J Clin Oncol.

[10] NCCN guidelines for patients with Waldenström macroglobulinaemia. https://www.nccn.org/patients/guidelines/content/PDF/waldenstrom-patient.pdf. Accessed 26 Oct 2023.

[11] Kyle RA, Treon SP, Alexanian R, Barlogie B, Björkholm M, Dhodapkar M (2003). Prognostic markers and criteria to initiate therapy in Waldenstrom’s macroglobulinemia: consensus panel recommendations from the Second International Workshop on Waldenstrom’s Macroglobulinemia. Semin Oncol.

[12] Owen RG, Kyle RA, Stone MJ, Rawstron AC, Leblond V, Merlini G (2013). Response assessment in Waldenström macroglobulinaemia: update from the VIth International Workshop. Br J Haematol.

[13] Ishikawa T, Takeuchi M, Shimada K, Kubo K, Kondo T, Fujimoto K (2022). Efficacy and safety of zanubrutinib in Japanese patients with mature B-Cell malignancies. Blood.

[14] Sekiguchi N, Rai S, Munakata W, Suzuki K, Handa H, Shibayama H (2020). A multicenter, open-label, phase II study of tirabrutinib (ONO/GS-4059) in patients with Waldenström’s macroglobulinemia. Cancer Sci.

[15] Burger JA, Barr PM, Robak T, Owen C, Ghia P, Tedeschi A (2020). Long-term efficacy and safety of first-line ibrutinib treatment for patients with CLL/SLL: 5 years of follow-up from the phase 3 RESONATE-2 study. Leukemia.

[16] Coutre SE, Byrd JC, Hillmen P, Barrientos JC, Barr PM, Devereux S (2019). Long-term safety of single-agent ibrutinib in patients with chronic lymphocytic leukemia in 3 pivotal studies. Blood Adv.

[17] O’Brien S, Furman RR, Coutre S, Flinn IW, Burger JA, Blum K (2018). Single-agent ibrutinib in treatment-naive and relapsed/refractory chronic lymphocytic leukemia: A 5-year experience. Blood.

[18] Marostica E, Sukbuntherng J, Loury D, de Jong J, de Trixhe XW, Vermeulen A (2015). Population pharmacokinetic model of ibrutinib, a Bruton tyrosine kinase inhibitor, in patients with B cell malignancies. Cancer Chemother Pharmacol.

