# Supplementary material for: A phase 2, open-label study of ibrutinib plus rituximab in Japanese patients with Waldenstrom’s macroglobulinemia
Source: Int J Hematol. 2024 Apr 10;120(1):80–90. doi: 10.1007/s12185-024-03761-9 (PMC11229445; doi:10.1007/s12185-024-03761-9)

**Supplementary material**

**Supplementary Table 1. Summary of Pharmacokinetics Parameters of ibrutinib and PCI-45227.**

|  | **Ibrutinib** | **PCI-45227** |
| --- | --- | --- |
| **Ibrutinib plus Rituximab /Week4 /Day1** | | |
| C_max_ (ng/mL), N, mean (SD) | 15  122 (149) | 15  87.2 (57.3) |
| T_max_ (hr), N | 15 | 15 |
| Mean (SD) | - | - |
| Median (range) | 3.62 (0.8, 4.22) | 3.75 (1.8, 5.0) |
| T_last_ (hr), N | 15 | 15 |
| Mean (SD) | - | - |
| Median (range) | 23.8 (5.0, 26.9) | 24.1 (23.1, 27.4) |
| AUC_24_ (ng*hr/mL), N, mean (SD) | 12  763 (834) | 11  1152 (808) |
| AUC_last_ (ng*hr/mL), N, mean (SD) | 15  836 (1115) | 15  1075 (768) |
| T_1/2_, term (hr), N, mean (SD) | 4  4.7 (1.2) | 3  8.7 (2.7) |
| λz (1/hr), N, mean (SD) | 4  0.2 (0.03) | 3  0.1 (0.03) |
| Metabolite/Parent Ratio C_max_ (Ratio), N, mean (SD) | 15  1.2 (0.9) | |
| Metabolite/Parent Ratio AUC_24_ (Ratio), N, mean (SD) | 11  1.8 (0.9) | |
| Metabolite/Parent Ratio AUC_last_ (Ratio), N, mean (SD) | 15  3.0 (3.1) | |

Dose-normalized C_max_, AUC_last_, and AUC_24_ will be calculated to a 420 mg ibrutinib dose.

Abbreviations: C = concentration, T = time, AUC = area under the curve, SD = standard deviation.

**Supplementary Table 2. Symptoms of WM^a^**

| Constitutional symptoms |  |
| --- | --- |
| Unintentional weight loss ≥10% within the previous 6 months  prior to screening | 2 (12.5%) |
| Fevers higher than 38.0°C for 2 or more weeks prior to screening without evidence of infection | 0 |
| Night sweats for more than 1 month prior to screening without evidence of infection | 1 (6.3%) |
| Clinically relevant fatigue which is not relieved by rest due to WM | 8 (50.0%) |
| Symptomatic hyperviscosity or serum viscosity levels >4.0 centipoises | 3 (18.8%) |
| Lymphadenopathy which is either symptomatic or bulky (>=5 cm in maximum diameter) | 0 |
| Symptomatic hepatomegaly or splenomegaly or organ tissue infiltration | 2 (12.5%) |
| Peripheral neuropathy due to WM | 1 (6.3%) |
| Symptomatic cryoglobulinemia | 0 |
| Cold agglutinin anemia | 0 |
| IgM related immune hemolytic anemia and/or thrombocytopenia | 0 |
| Nephropathy related to WM | 0 |
| Amyloidosis related to WM | 0 |
| Hemoglobin ≤10 g/dL | 10 (62.5%) |
| Platelet count <100,000 cells/uL | 0 |
| Serum monoclonal protein >5 g/dL, with or without overt clinical symptoms | 0 |
| Other | 0 |

^a^Subjects may appear in more than one category. Percentages calculated with the number of subjects in all treated analysis set as denominator.

Abbreviations: IGM = Immunoglobulin M, WM = waldenstrom’s macroglobulinemia.

**Supplementary Figure 1. Patient Disposition**


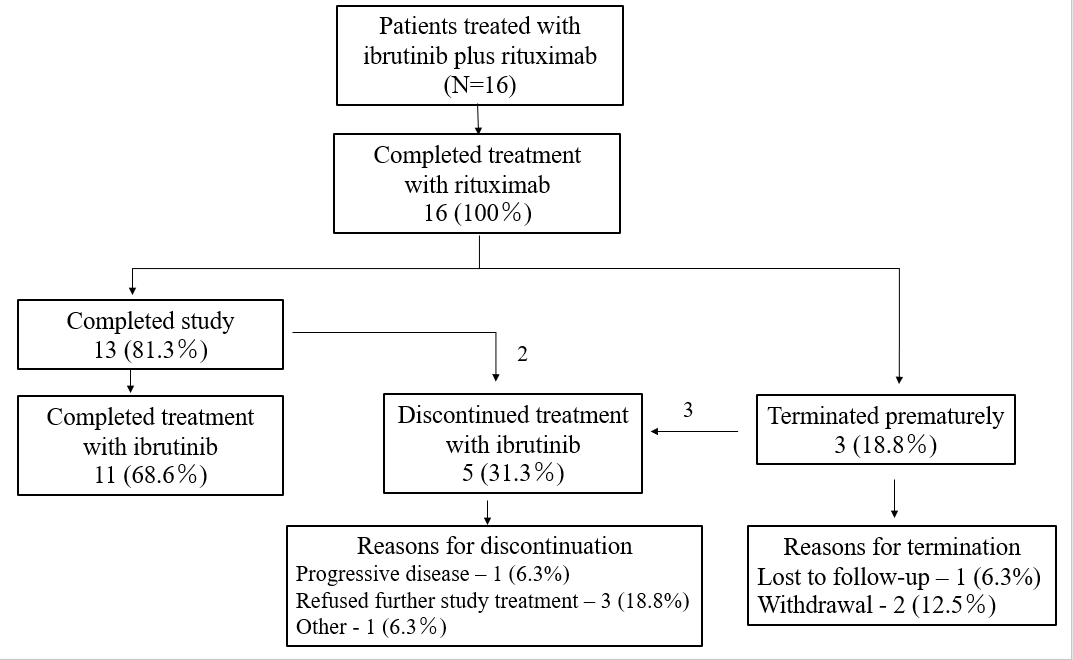

Supplement: Supplementary file 1 — Supplementary file1 (DOCX 74 KB) [file 12185_2024_3761_MOESM1_ESM.docx]
